# Complete Genome Sequence of Type Strain *Pasteurella multocida* subsp. *multocida* ATCC 43137

**DOI:** 10.1128/genomeA.01070-14

**Published:** 2014-10-23

**Authors:** K. W. Davenport, H. E. Daligault, T. D. Minogue, K. A. Bishop-Lilly, D. C. Bruce, P. S. Chain, S. R. Coyne, K. G. Frey, J. Jaissle, G. I. Koroleva, J. T. Ladner, C. C. Lo, G. F. Palacios, C. L. Redden, M. B. Scholz, H. Teshima, S. L. Johnson

**Affiliations:** a Los Alamos National Laboratory (LANL), Los Alamos, New Mexico, USA; b Diagnostic Systems Division (DSD), United States Army Medical Research Institute of Infectious Diseases (USAMRIID), Fort Detrick, Maryland, USA; c Naval Medical Research Center (NMRC-Frederick), Fort Detrick, Maryland, USA; d Henry M. Jackson Foundation, Bethesda, Maryland, USA; e Center for Genome Sciences (CGS), USAMRIID, Fort Detrick, Maryland, USA

## Abstract

Soft-tissue infection by *Pasteurella multocida* in humans is usually associated with a dog- or cat-related injury, and these infections can become aggressive. We sequenced the type strain *P. multocida* subsp. *multocida* ATCC 43137 into a single closed chromosome consisting of 2,271,840 bp (40.4% G+C content), which is currently available in the NCBI GenBank under the accession number CP008918.

## GENOME ANNOUNCEMENT

*Pasteurella* is a bacterial genus composed mostly of commensals or zoonotic pathogens. However, generally as a result of scratches, bites, or licks from infected animals, several species can cause human infection. Members are small Gram-negative nonmotile coccobacilli. The species comprises three subspecies (*gallicida*, *multocida*, and *septica*), which are differentiated based on biochemical assays or PCR fingerprints (1). *P. multocida* possesses several potential virulence factors such as iron acquisition proteins, capsule lipopolysaccharide (LPS), and hemagglutinin (2, 3). *P. multocida* subsp. *multocida* ATCC 43137 is the type strain, exhibits a type-A LPS, and is commonly used as a reference strain in pathogenicity studies.

High-quality genomic DNA was extracted from a 100-ml culture of a purified isolate according to manufacturer’s directions using QIAgen Genomic-tip 500. DNA was sequenced using Illumina technology. Genome assembly was performed by the Los Alamos National Laboratory (LANL) Genome Science Group, and 300-fold 100-bp paired-end (270 ± 30 bp insert) Illumina data (4) were assembled in Newbler (version 2.6), Velvet (version 1.2.08) (5), and AllPaths (version 42298) (6). Consensus sequences from all assemblers were computationally shredded and assembled with a subset of read pairs from the long-insert library using Phrap (version SPS-4.24) (7, 8). The resulting assembly was brought to closed and finished status through both manual and computational finishing efforts using Consed (9) and in-house scripts. The assembled genome sequence was corrected by mapping Illumina reads back to the final consensus sequences using Burrows-Wheeler Alignment (BWA) (10), SAMtools (11) and in-house scripts. Annotations were completed at LANL using an automated system utilizing the Ergatis workflow manager (12) and in-house scripts.

The 2.27-Mbp (40.4% G + C content) complete assembly of *P. multocida* subsp. *multocida* ATCC 43137 assembly includes 2,076 coding sequences, 19 rRNA, and 58 tRNA sequences. Preliminary review of the annotated genome indicates resistance genes for multiple toxic metals, iron acquisition, hemagglutinin, and genes for LPS production.

### Nucleotide sequence accession number.

The complete genome assembly was deposited in GenBank under the accession number CP008918.
